# Enhanced Antifibrinolytic Efficacy of a Plasmin-Specific Kunitz-Inhibitor (60-Residue Y11T/L17R with C-Terminal IEK) of Human Tissue Factor Pathway Inhibitor Type-2 Domain1

**DOI:** 10.3390/jcm9113684

**Published:** 2020-11-17

**Authors:** Kanagasabai Vadivel, Anne K. Zaiss, Yogesh Kumar, Frank M. Fabian, Ayman E. A. Ismail, Mark A. Arbing, Wallace G. Buchholz, William H. Velander, S. Paul Bajaj

**Affiliations:** 1 Department of Orthopedic Surgery, David Geffen School of Medicine, University of California, Los Angeles, CA 90095, USA; Kvadivel@mednet.ucla.edu (K.V.); AZaiss@mednet.ucla.edu (A.K.Z.); dr.yogesh.kumar@gmail.com (Y.K.); 2 Department of Chemical and Biomolecular Engineering, University of Nebraska, Lincoln, NE 68588, USA; frank.fabian@wallawalla.edu (F.M.F.); ismailo80@yahoo.com (A.E.A.I.); buchholzwg@gmail.com (W.G.B.); wvelander2@unl.edu (W.H.V.); 3 Chemistry Department, Walla Walla University, College Place, WA 99324, USA; 4 Protein Expression Technology Center, UCLA-DOE Institute, University of California, Los Angeles, CA 90095, USA; marbing@mbi.ucla.edu; 5 Molecular Biology Institute, University of California, Los Angeles, CA 90095, USA

**Keywords:** aprotinin, ε-aminocaproic acid, tranexamic acid, antifibrinolytics, thromboelastography

## Abstract

Current antifibrinolytic agents reduce blood loss by inhibiting plasmin active sites (e.g., aprotinin) or by preventing plasminogen/tissue plasminogen activator (tPA) binding to fibrin clots (e.g., ε-aminocaproic acid and tranexamic acid); however, they have adverse side effects. Here, we expressed 60-residue (_NH2_NAE…IEK_COOH_) Kunitz domain1 (KD1) mutants of human tissue factor pathway inhibitor type-2 that inhibit plasmin as well as plasminogen activation. A single (KD1-L17R-K_COOH_) and a double mutant (KD1-Y11T/L17R- K_COOH_) were expressed in *Escherichia coli* as His-tagged constructs, each with enterokinase cleavage sites. KD1-Y11T/L17R-K_COOH_ was also expressed in *Pichia pastoris*. KD1-Y11T/L17R-K_COOH_ inhibited plasmin comparably to aprotinin and bound to the kringle domains of plasminogen/plasmin and tPA with *K_d_* of ~50 nM and ~35 nM, respectively. Importantly, compared to aprotinin, KD1-L17R-K_COOH_ and KD1-Y11T/L17R-K_COOH_ did not inhibit kallikrein. Moreover, the antifibrinolytic potential of KD1-Y11T/L17R-K_COOH_ was better than that of KD1-L17R-K_COOH_ and similar to that of aprotinin in plasma clot-lysis assays. In thromboelastography experiments, KD1-Y11T/L17R-K_COOH_ was shown to inhibit fibrinolysis in a dose dependent manner and was comparable to aprotinin at a higher concentration. Further, KD1-Y11T/L17R-K_COOH_ did not induce cytotoxicity in primary human endothelial cells or fibroblasts. We conclude that KD1-Y11T/L17R-K_COOH_ is comparable to aprotinin, the most potent known inhibitor of plasmin and can be produced in large amounts using *Pichia*.

## 1. Introduction

In severe trauma and during major surgical procedures, such as cardiac surgery, the fibrinolytic system is hyperactivated, resulting in severe hemorrhaging [1,2,3]. Extensive bleeding poses significant mortality risks and costs in battlefields, accidents and hospital settings. Uncontrolled bleeding is the leading cause of preventable death in trauma and often leads to the need for extensive blood transfusions during surgeries [4,5]. Antifibrinolytics, by inhibiting fibrinolysis, and thereby fibrin degradation products, reduce transfusion requirements [6,7]. Aprotinin (bovine pancreatic trypsin inhibitor, BPTI), a potent inhibitor of the plasmin active site, had been the leading antifibrinolytic agent to reduce blood loss during cardiac surgery and extremity trauma [8]. However, its use has been linked to severe side effects, such as kidney damage, myocardial infarction, and strokes [9,10]. Furthermore, aprotinin is of bovine origin, and its anaphylactic potential is a major concern [11]. For these reasons, it was temporarily removed from the clinical market in 2008 [12]. The currently approved therapeutic agents, tranexamic acid (TXA) and ε-aminocaproic acid (EACA), are lysine analogues, which avert the binding of plasminogen and tissue plasminogen activator (tPA) to the fibrin clot [13,14]. As a result, localized activation of plasminogen to plasmin is prohibited and fibrinolysis is prevented. However, EACA and TXA are not as effective as aprotinin in reducing blood loss [15]. Furthermore, like aprotinin, they also cause kidney failure [16], and recent evidence indicates that TXA, and to a lesser extent EACA, are associated with a significant incidence of seizures [16,17]. Therefore, an improved antifibrinolytic agent is needed that is devoid of adverse effects of aprotinin and lysine analogs.

Several active site plasmin inhibitors have been reported in the literature [18,19,20,21,22,23,24,25], but the development stages of most of them are unknown. Textilinin-1 (Q8008), the Kunitz domain plasmin active site inhibitor from *Pseudonaja textilis* [19,26], is under preclinical development. Q8008 inhibits plasmin with 10- to 15-fold weaker affinity than aprotinin, but it also inhibits kallikrein poorly [19,26]. In a mouse tail bleeding model, Q8008 was reported to be as effective as aprotinin at reducing blood loss [26]. However, since Q8008 is derived from snake venom, it can cause anaphylactic response in humans similar to that observed for aprotinin. Moreover, using the scaffold of sunflower trypsin inhibitor-1, a very potent cyclic peptide active site inhibitor of plasmin was designed with 0.05 nM *Ki*; it has been proposed as a candidate for drug development [25]. Additionally, allosteric synthetic fibrinolytic inhibitors have been proposed to reduce perioperative bleeding, but they are in very early stages of development [27,28]. Furthermore, a very potent plasmin inhibitor (DX-1000) is being developed as an antineoplastic agent instead of as an antifibrinolytic agent [29].

Notably, when aprotinin was banned in 2008, the pharmacologic agent ecallantide (DX-88), which inhibits both kallikrein and plasmin, was clinically evaluated [30]. This study was terminated prematurely due to an increased mortality observed in the ecallantide arm. Another agent, MDCO2010, which inhibits plasmin, factor (F) Xa, FXIa and activated protein C (APC), was also clinically evaluated [31]. This study was terminated prematurely as well due to an increased number of serious adverse events in the treatment groups. The causes of the safety issues are under investigation.

In the current study, we describe an antifibrinolytic agent which inhibits plasmin with comparable potency to aprotinin, but which is a very weak inhibitor of kallikrein. It was designed using the Kunitz domain 1 (KD1) of human tissue factor pathway inhibitor type-2 (TFPI-2) as a scaffold. The new 60-residue plasmin inhibitor, KD1-Y11T/L17R-K_COOH_, has one additional mutation with a different C-terminal lysine (IEK_COOH_), compared to the earlier heterogeneous single mutant KD1-L17R with C-terminal IEKVPK (designated KD1-L17R-K_T_) [32]. KD1-Y11T/L17R-K_COOH_ inhibits plasmin better than the current single mutant KD1-L17R-K_COOH_, and in addition to plasmin, it also binds to the kringle domains of plasminogen and tPA with 35 to 50 nM dissociation constants. It is anticipated that at a therapeutic dose of 2 µM in plasma, the lowest Hammersmith regime of aprotinin [33], KD1-Y11T/L17R-K_COOH_ will inhibit fibrinolysis effectively by inhibiting the plasmin active site as well as by blocking the binding of plasminogen and tPA to the fibrin clot. Thus, KD1-Y11T/L17R-K_COOH_ appears to be a promising candidate to replace aprotinin in clinical settings. Experimental details comparing aprotinin with KD1-Y11T/L17R-K_COOH_ are presented herein. Moreover, modeling was used to evaluate the effect of Tyr11 to Thr mutation as well as IEK at the C-terminus in the TFPI-2 KD1 inhibitor scaffold. Structural information gained from such modeling to delineate the enhanced antifibrinolytic activity of the mutants is discussed.

## 2. Experimental Section

### 2.1. Materials

*Escherichia coli* (*E. coli*) strain BL21(DE3) pLysS and pET28a expression vector were obtained from Novagen Inc. (Madison, WI, USA). Amicon centrifugal filter devices (3000 Mr cutoff) were purchased from Millipore (Bedford, MA, USA). QSepharose FF, Superdex 200, and His-Trap HP columns were obtained from Amersham Biosciences. Diisopropylfluorophosphate (DFP) was from Calbiochem (San Diego, CA, USA). TXA, EACA, kanamycin and isopropyl thiogalactopyranoside (IPTG) were obtained from Sigma (St. Louis, MO, USA). Caspase-Glo 3/7 Assay kit and CellTox™ Green Cytotoxicity Assay kit were from Promega (Madison, WI, USA). Purified human FXIa, thrombin (IIa) and plasmin were purchased from Hematologic Technologies Inc (Essex Junction, VT, USA). Plasma kallikrein (pKLK) was from Enzyme Research Laboratories (South Bend, IN, USA). Alteplase (tPA) was purchased from Genentech (South San Francisco, CA, USA). Recombinant enterokinase was from Novogen, EMD Chemicals (San Diego, CA, USA). Normal pooled plasma (NPP) was purchased from George King Bio-Medical Inc. (Overland Park, Kansas). δPlasmin (recombinant plasmin containing the protease domain and the first kringle domain) was obtained from Dr. Victor Marder (University of California, Los Angeles, CA, USA) and taxol was kindly provided by Dr. Zhenfeng Duan (University of California, Los Angeles, CA, USA). Aprotinin (BPTI) was received from ZymoGenetics (Seattle, WA, USA), and human factor VIIa (FVIIa) was prepared as described previously [34]. Soluble tissue factor (sTF, residues 1-219) was obtained from Tom Girard (Washington University, St. Louis, MO, USA). Plasmin substrate S-2251 (H-D-Val-Leu-Lys-*p*-nitroanilide), pKLK, and FXIa substrate S-2366 (pyroGlu-Pro-Arg-*p*-nitroanilide), and FVIIa substrate S-2288 (H-DIle-Pro-Arg-*p*-nitroanilide) were obtained from Diapharma Inc (West Chester, OH, USA). Fresh normal human citrated blood was bought from Nebraska Medical Center, Omaha. Partial thromboplastin time (PT) and activated partial thromboplastin time (aPTT) were normal for each blood donor.

### 2.2. Expression and Purification of KD1-L17R-K_COOH_ and KD1-Y11T/L17R-K_COOH_ in E. coli

**Figure 1:**
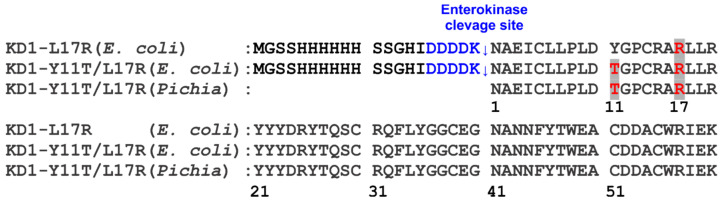
The expressed sequences of human TFPI-2 KD1 single mutant (KD1-L17R-K_COOH_) in *E. coli* and double mutant (KD1-Y11T/L17R-K_COOH_) in *E.coli* and *Pichia pastoris*. The down arrows indicate the enterokinase cleavage site introduced to remove the His-tag. The mutated residues Tyr11Thr and Leu17Arg are marked in red and the enterokinase cleavage sequence introduced is marked in blue. Residue 1 is numbered according to the BPTI-Kunitz domain numbering and corresponds to the amino acid 10 in the TFPI-2 Kunitz domain1 sequence.

The cDNA sequences of KD1-L17R-K_COOH_ and KD1-Y11T/L17R-K_COOH_ with C-terminal IEK were cloned and overexpressed as amino-terminal His_6_-tagged fusion proteins in *E. coli* strain BL21(DE3) pLysS using the T7 promoter system. The recombinant plasmid derived from pET28a, containing a His_6_ leader sequence followed by an enterokinase cleavage site and the cDNA encoding the KD1-L17R-K_COOH_ or KD1-Y11T/L17R-K_COOH_, was prepared according to standard procedures [35]. The sequences of the constructs expressed are given in Figure 1. The His_6_-tagged KD1-L17R-K_COOH_ and KD1-Y11T/L17R-K_COOH_ were expressed in *E. coli* grown in Luria broth containing 15 mg/liter kanamycin and induced at 37 °C with 1 mM IPTG at mid-log phase (A_600_ ~0.9) for 5–6 h at 37 °C. The His_6_-tagged KD1-L17R-K_COOH_ and KD1-Y11T/L17R-K_COOH_ were purified from the inclusion bodies using a nickel-charged His-Trap column. The His-Trap purified proteins were refolded using the reduced and oxidized glutathione system and further purified using Q-Sepharose FF column as described previously [32,36].

### 2.3. KD1-Y11T/L17R-K_COOH_ Clone Construction and Expression in Pichia pastoris

*Pichia pastoris* strain X-33 and the secretion expression vector pPICZαA were purchased from Invitrogen (San Diego, CA, USA). KD1-Y11T/L17R-K_COOH_ cDNA corresponding to the amino acid sequence (Figure 1) was synthesized by IDT (Coralville, IA, USA). The cDNA was amplified by PCR, and the product was linearized and subcloned into XhoI and NotI restriction sites of pPICZαA. Further vector amplification was carried out in DH5α competent cells. Extracted cDNA was introduced into *P. pastoris* X-33 via electroporation with a Bio-Rad Gene Pulser electroporator. The transformants were plated on YPD plates supplemented with 500 µg zeocin/mL. Colonies were evaluated by SDS-PAGE for KD1-Y11T/L17R-K_COOH_ expression in BMM medium. Fermentation inoculation shake flasks were prepared using buffered minimal glycerol medium (BMGY) pH 6.0. First, a single colony expressing KD1-Y11T/L17R-K_COOH_ was inoculated into 50 mL for 12 h, and 0.5 mL of the resulting culture was transferred to 300 mL BMGY pH 6.0 for 20 h. The latter was then inoculated into a 15 L NLF BioEngineering Bioreactor (Wald, Switzerland) containing 3 L Basal Salts Medium (BSM) pH 5.0. Protein expression was induced with methanol and carried out for 48 h at 30 °C, pH 5.0 and 40% dissolved oxygen. Fermentation broth was centrifuged at 7200 rpm at 4 °C. Supernatant containing KD1-Y11T/L17R-K_COOH_ was collected and stored at −30 °C until further processing.

### 2.4. KD1-Y11T/L17R-K_COOH_ Purification from Pichia pastoris

One hundred milliliters of fermentation supernatant was adjusted to pH 8.5 and centrifuged for 5 min at 1500 RFC at room temperature. Then, the supernatant was collected and adjusted to pH 3.0 and mixed with 0.5 mL Titron X-100 for 30 min. Urea was added up to a final concentration of 4 M and incubated for 2.5 h at room temperature. After incubation, the solution was diluted to a final conductivity of 12 mS. Purification was carried out using Biocad Vision workstation at constant flow rate of 120 cm/h. Sample was loaded on to a SP-Sepharose (GE Healthcare Bio-Sciences Pittsburgh, PA) column previously equilibrated with 50 mM Phosphate buffer pH 2.8 (wash buffer). Then, the column was washed with two column volume wash buffer, and protein was eluted with 50 mM phosphate buffer, pH 2.8, containing 1.0 M NaCl. The fractions containing KD1-Y11T/L17R-K_COOH_ were pooled and supplemented with L-Arginine to 0.5 M and with mannitol to 7%. After 1 h of incubation at room temperature, it was dialyzed against 10 mM phosphate buffer, pH 8.0, using 3.5 kDa MW cutoff membrane dialysis tubing (Spectrum, Palisades Park, NJ, USA).

### 2.5. SDS-PAGE

SDS-PAGE was performed using the Laemmli buffer system [37]. The acrylamide concentration used was 15%, and the gels were stained with Coomassie Brilliant Blue dye.

### 2.6. Protease Inhibition Assay

All reactions were carried out in TBS, pH 7.5 (50 mM Tris-HCl, containing 100 mM NaCl), containing 0.1 mg bovine serum albumin/mL (TBS/BSA) and 2 mM Ca^2+^ (TBS/BSA/Ca^2+^, pH 7.5). Each enzyme (plasmin, pKLK, FXIa or FVIIa/sTF) was incubated with various concentrations (10^−1^ to 2 × 10^3^ nM) of KD1-WT, KD1-L17R-K_COOH_, KD1-Y11T/L17R-K_COOH_ or aprotinin (BPTI) for 1 h at room temperature in a 96-well microtitration plate (total volume 100 µL/well). A synthetic substrate (5 µL) appropriate for each enzyme was then added to a final concentration of 1 *K_M_*, and residual amidolytic activity was measured in a *V_max_* kinetic microplate reader (Molecular Devices, San Jose, CA, USA). The inhibition constant K_i_^*^ was determined using the nonlinear regression data analysis program, Grafit. Data for aprotinin, KD1-WT, KD1-L17R-K_COOH_ and KD1-Y11T/L17R-K_COOH_ were analyzed with an equation for a tight-binding inhibitor (Equation (1)), where *v_i_* and *v*_0_ are the inhibited and uninhibited rates, respectively, and [I]_0_ and [E]_0_ are the total concentrations of inhibitor and enzyme, respectively [38,39].

(1) vi=v0((Ki*+[I]0+[E]0)2−4[I]0[E]0)12−(Ki*+[I]0−[E]0)2[E]0

*K_i_* values were obtained by correcting for the effect of substrate according to Beith [38], using Equation (2), where [*S*] is substrate concentration and *K_M_* is specific for each enzyme.

(2) $$K_{i}=\frac{K_{i}^{*}}{\left(1+\left[S\right]/K_{M}\right)}$$

### 2.7. Preparation of DIP-δplasmin

Active-site blocked δplasmin was generated by treating δplasmin with equal volumes of 1 M Tris-HCl, pH 8.0 and 1 M DFP (final concentration of 1 mM DFP) at room temperature for 20 min, followed by incubation on ice for several hours. Additional equal volumes of 1M Tris-HCl, pH 8.0 and 1M DFP (final concentration of 2 mM) were added and the reaction was incubated at room temperature for 20 min and then over night at 4 °C. The DFP inhibited δplamsin (DIP-δplasmin) was dialyzed against 20 mM HEPES pH 7.5, containing 150 mM NaCl and assayed for residual activity using S-2251 synthetic substrate hydrolysis. Based upon the residual activity, >99% of the δplasmin was inactivated. DIP-δplasmin, when analyzed using SDS-PAGE, revealed no protein degradation.

### 2.8. KD1-Y11T/L17R-K_COOH_ Binding to tPA and DIP-δplasmin Using Surface Plasmon Resonance (SPR)

Binding studies were performed on a Biacore T100 flow biosensor (Biacore, Uppsala, Sweden) at 25 °C. DIP-δplasmin (~98% purity using SDS-PAGE) or tPA (>98% purity using SDS-PAGE) was immobilized on carboxymethyl-dextran flow cell (CM5 sensor chips, GE Healthcare) using amine-coupling chemistry. Flow cell surfaces were activated with a mixture of 1-ethyl-3-(3-dimethylaminopropyl) carbodiimide and N-hydroxysulfosuccinimide for 5 min (flow rate 10 μL/min), after which the protein (20 μg/mL in 10 mM sodium acetate, pH 5.5) was placed upon the surface. Unreacted sites were blocked for 5 min with 1 M ethanolamine. The analyte KD1-Y11T/L17R-K_COOH_ (100 to 2000 nM) was perfused through flow cells in HBS-P buffer (20 mM HEPES, pH 7.4, 100 mM NaCl, 0.005% (*v*/*v*) P20) at 10 μL/minute for 6 min. After changing to HBS-P buffer without the protein, analyte dissociation was monitored for 10 min. Flow cells were regenerated with HBS-P containing 20 mM EACA. Data were corrected for nonspecific binding by subtracting signals obtained with the analyte infused through a flow cell without the coupled protein. Binding was analyzed with BIAevaluation software (Biacore) using a 1:1 binding model. *K_d_* values were calculated from the quotient of the derived dissociation (*k_d_*) and association (*k_a_*) rate constants.

### 2.9. Fibrinolysis (Clot Lysis) Assay

The method of Sperzel and Huetter [40] was followed with minor modifications as outlined earlier [32,41]. Briefly, IIa was used to initiate fibrin formation in NPP and the lysis of the formed clot (fibrinolysis) was induced by simultaneous addition of tPA. Clot formation and lysis were monitored with a Molecular Devices microplate reader (SPECTRAmax 190) measuring the optical density at 405 nm. Briefly, 10 µL of each test compound (KD1-L17R-K_COOH_, KD1-Y11T/L17R-K_COOH_, aprotinin) or saline control was added to 240 µL of NPP. Two hundred twenty-five microliters of this mixture was then added to 25 µL IIa and tPA in TBS/BSA containing 25 mM CaCl_2_. In the 250 µL final volume, the concentration of IIa was 0.15 µg/mL and that of tPA was 1 µg/mL. Under control conditions (zero tPA and zero test compound), OD_405_ increased immediately indicating clotting followed by an extremely slow decrease, representing fibrinolysis. As clotting was almost complete after 5 min, fibrinolysis induced by tPA was evaluated as a relative decrease of OD_405_ up to 60 min. KD1-L17R-K_COOH_ was tested at final concentrations from 0.5 µM to 5 µM, while KD1-Y11T/L17R-K_COOH_ and aprotinin were tested at final concentrations from 0.5 µM to 3 µM.

### 2.10. Thromboelastography

The effect of different concentrations of KD1-WT, KD1-L17R-K_COOH_, KD1-Y11T/L17R-K_COOH_, aprotinin or EACA on fibrinolysis was evaluated with thromboelastography (TEG) using a TEG 5000 Thrombelastograph (Haemonetics Corp, Braintree, MA, USA). Each clot formation/lysis assay contained 300 µL of citrated whole blood, plasmin (1.5 µM final concentration), CaCl_2_ (10 mM final concentration) and various concentrations of each antifibrinolytic agent in Ringer’s solution to make the final volume to 360 µL. Plasmin and CaCl_2_ were added last to initiate simultaneous clotting and fibrinolysis. A 1.5 µM plasmin concentration was chosen based on the plasmin effect on the clot strength and lysis. Each experiment was performed for 180 min to establish the LY60 value. The thromboelastograph was calibrated each day, and each inhibitor concentration was tested in duplicate. TEG Analytical Software (version 4.2.2; Haemonetics Corporation, Braintree, MA, USA) was used to calculate the time to clot initiation (R), maximal clot strength (maximal amplitude (MA), which was directly related to the shear elastic modulus strength, G), and percent lysis 60 min after MA (LY60) [42].

### 2.11. Cytotoxicity Assays

#### 2.11.1. Cells and Culture Conditions

Primary human pooled umbilical vein endothelial cells (HUVEC) were obtained from ATCC. The cells were maintained in Vascular Cell Basal Medium (ATCC), supplemented with the Endothelial Cell Growth Kit-BBE (ATCC) and Penicillin-Streptomycin-Amphotericin B (ATCC). Primary human dermal skin fibroblasts were obtained from LONZA and maintained in Fibroblast Basal Medium (FBM^TM^, LONZA, Basel, Switzerland), supplemented with a cocktail of growth factors, fetal bovine serum and antibiotics (FGM^TM^-2 SingleQuots^TM^, LONZA). All cells were maintained in a humidified 5% CO_2_ atmosphere at 37 °C and were passaged once they reached 80% confluence. All experiments were performed with cells in the logarithmic growth phase.

#### 2.11.2. Antifibrinolytic Agents (KD1-Y11T/L17R-K_COOH_, Aprotinin, EACA and TXA)

Stock solutions of antifibrinolytic agents were prepared in phosphate buffer. For toxicity studies, cells were seeded into 96- or 24-well cell culture plates at 3500 cells/cm^2^, and were used for experiments once they reached 80% confluence. Cells were treated with antifibrinolytic agents for 24 h at the following concentrations: aprotinin and KD1-Y11T/L17R-K_COOH_ at 0.1 µM, 1 µM, 10 µM and 30 µM; EACA at 1 mM, 5 mM, 20 mM, and 60 mM and TXA at 0.2 mM, 2 mM, 10 mM and 30 mM.

#### 2.11.3. Resazurin Reduction Assay

Resazurin reduction assay (Fisher Scientific) was used to evaluate the potential cytotoxicity of antifibrinolytic agents toward primary human endothelial cells and skin fibroblasts. The phosphate buffer that was used to dissolve the samples was included as negative control. The assay was based on the reduction of the nonfluorescent dye resazurin to the highly fluorescent resorufin by viable cells. The fluorescent signal is proportional to the number of live cells, since nonviable cells are unable to reduce the dye and do not produce fluorescent signals. Briefly, cells in 96-well cell culture plates were treated with different concentrations of antifibrinolytic compounds (as described above). After 24 h, resazurin reagent was added to each well and the plates were incubated at 37 °C for 4 h. Fluorescence was measured by the FLUOstar Omega Microplate Reader (BMG Labtech, Offenburg, Germany) using an excitation wavelength of 544 nm and an emission wavelength of 590 nm. Each assay was done in duplicate, with three replicates each. The viability was evaluated based on comparison with untreated cells.

#### 2.11.4. Caspase 3/7 Assay

The influence of antifibrinolytic agents on apoptosis in cells was detected using the Caspase-Glo 3/7 Assay kit (Promega). Caspases 3 and 7 were activated in cells that undergo apoptosis. The assay provided a luminogenic substrate for caspase 3 and 7. Enzymatic activity leads to luminescence, which is proportional to the amount of caspase activity present. Cells were seeded in 96-well plates and treated with antifibrinolytic agents or phosphate buffer (solvent control). Taxol was included as a positive control. After 24 h of treatment, caspase reagent was added to each well, mixed and incubated for 1 h at room temperature. Luminescence was measured using the FLUOstar Omega Microplate Reader (BMG Labtech).

#### 2.11.5. Cell Toxicity Assay

Cell toxicity and cell death were evaluated with the CellTox™ Green Cytotoxicity Assay (Promega). This assay measures changes in membrane integrity that occur as a result of cell death. The dye used in the system is excluded from viable cells but binds to DNA in compromised cells, which results in a fluorescent signal. We measured cell death in HUVEC and primary fibroblasts treated with antifibrinolytic agents at four concentrations (as indicated above) with triplicates per concentration in 24-well plates after 24 h of exposure. Hoechst (Thermo Fisher Scientific, Waltham, MA, USA) was used to stain all nuclei. Images of cells were captured using an inverted microscope (Nicon; Edipse T2000 TE). Green fluorescent cells (FITC filter) and Hoechst stained cells (DAPI filter) were counted using Image J software. Fluorescent cells were displayed as a percentage of all cells.

### 2.12. Statistical Methods

One-way analysis of variance (ANOVA) was used to compare the effect of antifibrinolytic agents in inhibiting fibrinolysis (KD1-WT, KD1-L17R-K_COOH_, KD1-Y11T/L17R-K_COOH_, aprotinin) in the plasma clot lysis assay. The *p* values for comparing any two means were computed using post hoc tests and adjusted for multiple comparisons using Tukey’s adjustment. For the TEG data, Levene’s F-test revealed that the homogeneity of variance was not met. As such, the Welch’s F-test was used and Games-Howell post hoc procedure was conducted to determine which pairs of the mean MA and mean LY60% levels differed significantly. For the cell toxicity assays, collected data sets were analyzed by ANOVA and individual groups were compared using the Student’s t-test. All experiments were replicated two or three times, with similar results. Quantitative values are reported as mean ± standard deviation (SD) or standard error of the mean (SEM), as indicated in the figure legends. Differences were considered statistically significant at *p* values of 0.05 or lower. All statistical analyses were performed using SPSS V27 (IBM Corp., Armonk, NY, USA).

### 2.13. Molecular Modeling

The crystal structures of µ-plasmin [43], plasminogen kringle domain1 [14] and wild-type KD1 [36] were used as templates to model the complexes of KD1-Y11T/L17R-K_COOH_ with µ-plasmin and with plasmin kringle domain1. The protocols for modeling these complexes have been described elsewhere [41,44]. Since the C-terminus residues are disordered in the wild-type KD1 crystal structure, we used the MODELLER program [45] to build this part of the KD1-Y11T/L17R-K_COOH_ molecule. The built models were further refined by subjecting to 1000-step minimization with the harmonic constraints of 10 kcal.mol^−1^. Å^−2^ using the AMBER program [46].

## 3. Results

### 3.1. Expression and Purification of KD1-L17R-K_COOH_ and KD1-Y11T/L17R-K_COOH_ in E. coli

**Figure 2:**
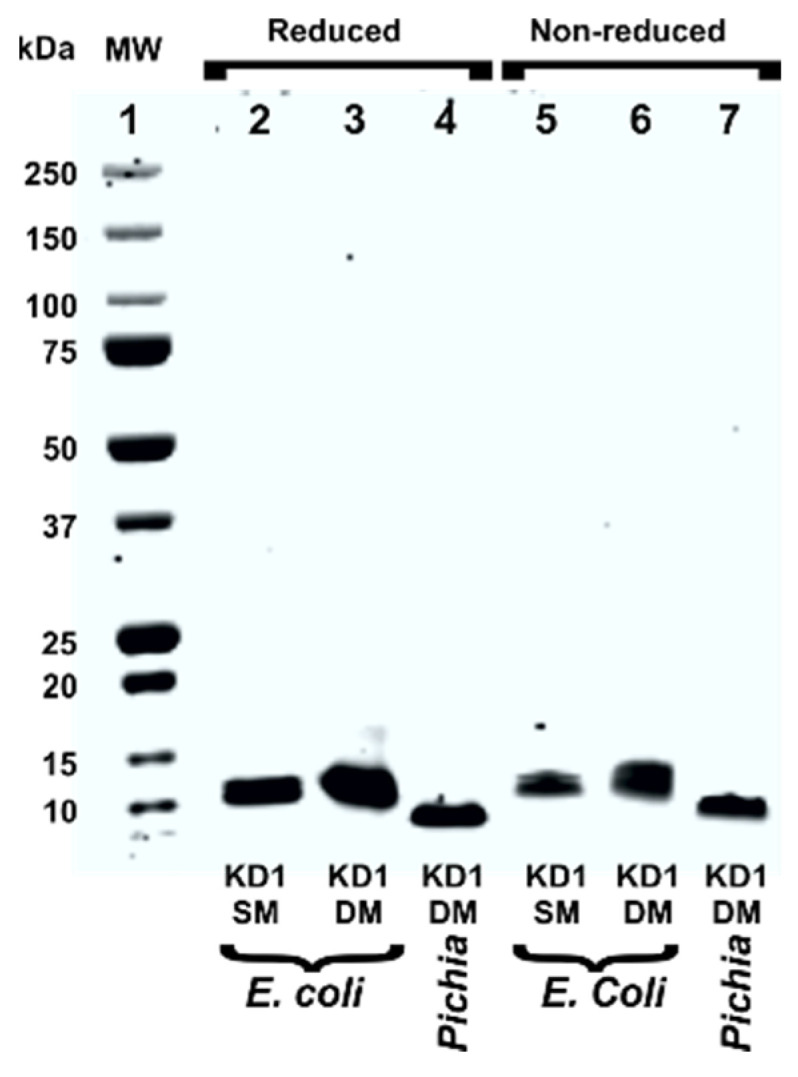
SDS-PAGE analysis of TFPI-2 KD1 single and double mutants. Lane 1, molecular weight (MW) markers; lane 2, reduced *E.coli* KD1-L17R-K_COOH_; lane 3, reduced *E.coli* KD1-Y11T/L17R-K_COOH_; lane 4, reduced *P. pastoris* KD1-Y11T/L17R-K_COOH_; lane 5, nonreduced *E. coli* KD1-L17R-K_COOH_; lane 6, nonreduced *E. coli* KD1-Y11T/L17R-K_COOH_; lane 7, nonreduced *P. pastoris* KD1-Y11T/L17R-K_COOH_. Five µg of protein was loaded in each lane. KD1SM, KD1-L17R-K_COOH_; and KD1DM, KD1-Y11T/L17R-K_COOH_.

The 60-residue His_6_-tagged KD1-L17R-K_COOH_ and KD1-Y11T/L17R-K_COOH_ were expressed in *E. coli* strain BL21 (DE3) pLysS with an enterokinase cleavage site (Figure 1). These constructs are 9-residues shorter at the N-terminus and 3-residues shorter at the C-terminus ending with IEK-COOH (Figure 1) as compared to the previously expressed KD1-L17R with IEKVPK at the C-terminus (designated KD1-L17R-K_T_) [41]. The fusion proteins were refolded and purified using Q-Sepharose FF column. The purified KD1 mutant proteins were incubated with enterokinase to remove the His_6_-tag; however, the cleavage was unsuccessful at 1:50 ratio of enzyme to substrate. The reason for the unsuccessful His_6_-tag removal could be the inhibition of enterokinase by KD1 mutants, similar to that described for the inhibition of enterokinase by aprotinin [47]. The SDS-PAGE analysis of purified KD1-L17R-K_COOH_ and KD1-Y11T/L17R-K_COOH_, each containing the enterokinase cleavage site and His_6_-tag at the NH_2_-terminus, is shown in Figure 2.

### 3.2. Expression and Purification of KD1-Y11T/L17R-K_COOH_ in P. pastoris

Since the His_6_-tag could not be removed by enterokinase in the *E. coli* expressed mutants, we expressed the 60-residue double mutant KD1-Y11T/L17R-K_COOH_ using *P. pastoris* and purified to homogeneity, as described in the Experimental section. Approximately 50 mg of KD1-Y11T/L17R-K_COOH_ was purified from 100 mL of culture media. The SDS-PAGE analysis of purified *P. pastoris* KD1-Y11T/L17R-K_COOH_ is shown in Figure 2. Note that the *P. pastoris* expressed KD1-Y11T/L17R-K_COOH_ was of slightly lower MW compared to the corresponding *E. coli* expressed KD1-Y11T/L17R-K_COOH_ containing the His_6_-tag and the enterokinase cleavage sequence (Figure 1).

### 3.3. Inhibition Profile of KD1-L17R-K_COOH_ and KD1-Y11T/L17R-K_COOH_

**Figure 3:**
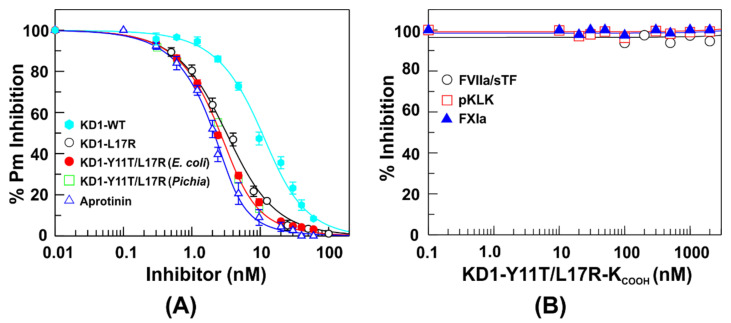
(**A**) Determination of equilibrium dissociation constants (*K_i_*) of *E. coli* expressed KD1-WT (IIa cleavage site, 32), *E. coli* expressed KD1-L17R-K_COOH_, KD1-Y11T/L17R-K_COOH_ with enterokinase cleavage sites, *P. pastoris* expressed KD1-Y11T/L17R-K_COOH_ and aprotinin with plasmin. The enzyme activity is expressed as the percent fractional activity (inhibited rate/uninhibited rate) at increasing inhibitor concentrations. The inhibition constants (*K_i_*) were determined using Equations (1) and (2) as outlined in the Experimental section. The data represent average of three experiments. The concentration of plasmin used was 3 nM. (**B**) Inhibition profile of KD1-Y11T/L17R-K_COOH_ with FVIIa/sTF, pKLK and FXIa. The concentration of FVIIa/sTF was 20 nM, whereas pKLK and FXIa were 1 nM each. No inhibition was observed up to 3 µM concentration of KD1-Y11T/L17R-K_COOH_ in the triplicate experiments performed.

Wild-type KD1 (KD1-WT) containing the IIa cleavage site inhibited plasmin with *K_i_* 6.0 ± 0.5 nM [32], Figure 3A. KD1-L17R-K_COOH_ with the IEK C-terminus containing the enterokinase cleavage site inhibited plasmin with *K_i_* 0.9 ± 0.1 nM similarly to previously described KD1-L17R-K_T_ with VPK C-terminal containing the IIa cleavage site [41]. Both *E. coli* and *P. pastoris* expressed KD1-Y11T/L17R-K_COOH_ inhibited plasmin (*K_i_* 0.59 ± 0.1) with similar affinities to aprotinin (*K_i_* 0.49 ± 0.1) (Figure 3A). The *K_i_* values for plasmin inhibition by each inhibitor are provided in Table 1. Thus, the enterokinase cleavage sequence and the His_6_-tag did not affect the inhibitory activity. Further, similar to KD1-L17R-K_T_ [32,41], KD1-Y11T/L17R-K_COOH_ (present study) weakly inhibited FVIIa/sTF, FXIa and pKLK with *K_i_* > 3 µM (Figure 3B).

### 3.4. KD1-Y11T/L17R-K_COOH_ Binding to DIP-δplasmin and tPA

**Figure 4:**
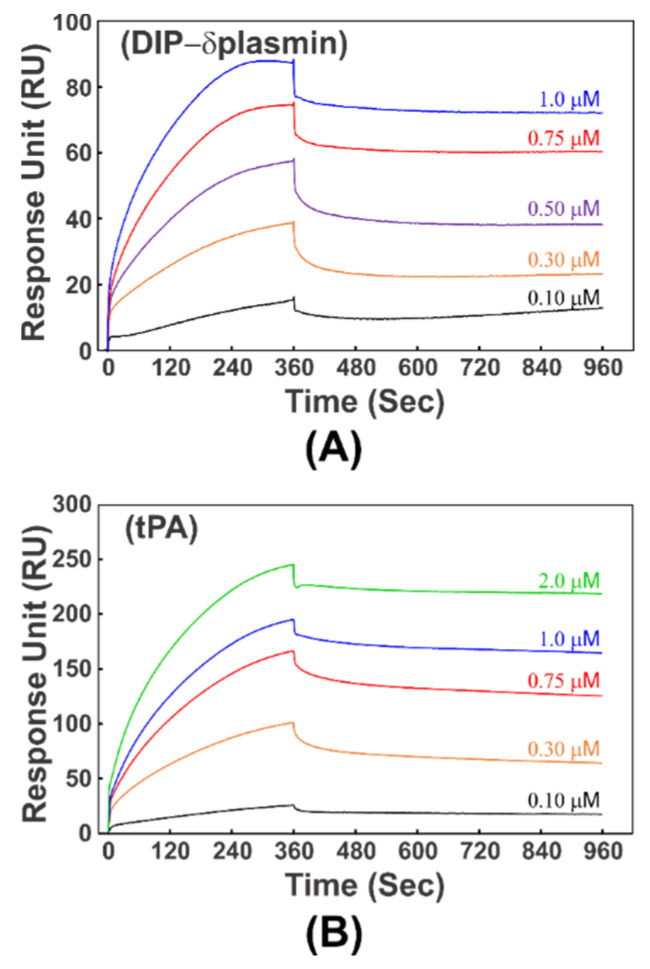
Interaction of KD1-Y11T/L17R-K_COOH_ with DIP-δplasmin and tPA as measured by SPR. (**A**) DIP-δplasmin binding to KD1-Y11T/L17R-K_COOH_. DIP-δplasmin was coupled to the CM5 chip by the amine coupling method, and an immobilization level of 734 response units (RU) was attained for the bound protein. Five concentrations (0.1 μM, 0.3 μM, 0.5 μM, 0.75 μM and 1 μM) of KD1-Y11T/L17R-K_COOH_ were used, and 6 min association and 10 min dissociation times (flow rate of 10 μL/min) were employed. Details are provided in the Experimental section. (**B**) tPA binding to KD1-Y11T/L17R-K_COOH_. The tPA was coupled to the CM5 chip, and an immobilization level of 1182 RU was attained for the bound protein. Five concentrations of KD1-Y11T/L17R-K_COOH_ (0.1 μM, 0.3 μM, 0.75 μM, 1 μM and 2 μM) were used. The analyte association and dissociation protocols were the same as in panel (**A**). Experiments in panel (**A**) and panel (**B**) were performed in duplicate. Each data set was then used to calculate *k_on_*, *k_off_* and *K_d_* values and to obtain the mean ± SD values provided in the text.

We used SPR to study the binding of KD1-Y11T/L17R-K_COOH_ to immobilized DIP-δplasmin (Figure 4A) and tPA (Figure 4B). The *k_on_* for binding of DIP-δplasmin to KD1-Y11T/L17R-K_COOH_ was 1.49 ± 0.3 × 10^3^ M^−1^s^−1^; *k_off_* was 7.13 ± 0.9 × 10^−5^ s^−1^, and the *K_d_* was 47.6 ± 7 nM. The *k_on_* for binding of tPA to KD1-Y11T/L17R-K_COOH_ was 2.91 ± 0.4 × 10^3^ M^−1^s^−1^; *k_off_* was 1.05 ± 0.7 × 10^−4^ s^−1^, and the *K_d_* was 35.4 ± 5 nM.

### 3.5. Fibrinolysis (Clot Lysis) Assay

**Figure 5:**
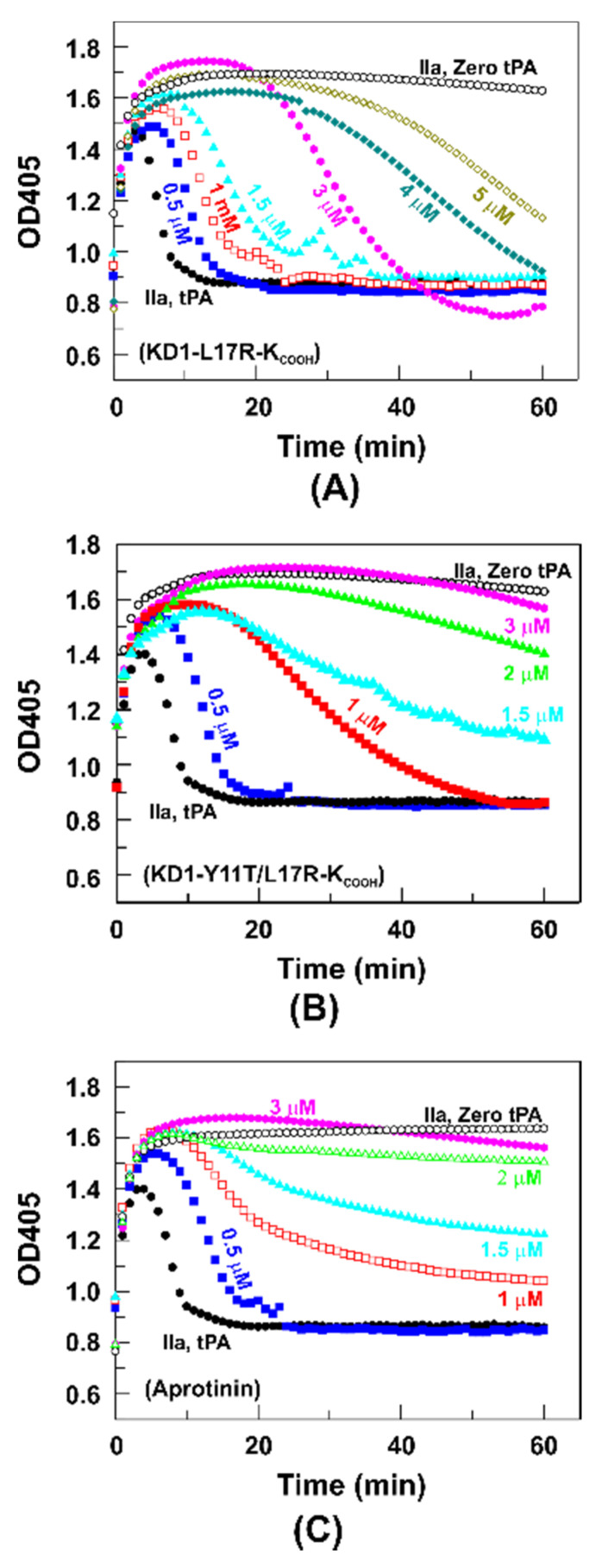
Effect of KD1-L17R-K_COOH_, KD1-Y11T/L17R-K_COOH_ and aprotinin on fibrinolysis in human NPP. IIa was added to NPP to initiate clot formation, which is associated with an increase in OD405 (curve **⚬**; IIa, Zero tPA in **A**–**C**). Simultaneous addition of tPA converted plasminogen to plasmin, which dissolved the fibrin clot completely within ~10 min, as indicated by an initial increase followed by a decrease in OD405 (curve **●**; IIa, tPA in **A**–**C**). Addition of KD1-L17R-K_COOH_, KD1-Y11T/L17R-K_COOH_ or aprotinin inhibited fibrinolysis in a dose-dependent manner. (**A**) Effect of KD1-L17R-K_COOH_; 0.5 µM (■), 1 µM (**□**), 1.5 µM (▲), 3 µM (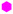), 4 µM (■) and 5 µM (**□**). (**B**) Effect of KD1-Y11T/L17R-K_COOH_; 0.5 µM (■), 1 µM (■), 1.5 µM (▲), 2 µM (▲) and 3 µM (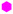). (**C**) Effect of aprotinin; 0.5 μM (■), 1 µM (**□**), 1.5 µM (▲), 2 µM (△) and 3 μM (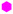).

These experiments were performed to compare the effectiveness of KD1-L17R-K_COOH_, KD1-Y11T/L17R-K_COOH_, and aprotinin at inhibiting tPA-induced plasma clot fibrinolysis. The addition of IIa to NPP caused fibrin formation, which was reflected by an increase in OD405 (curve IIa, Zero tPA, Figure 5A−C). The simultaneous addition of tPA caused initial clot formation followed by the dissolution of fibrin induced by tPA-mediated conversion of plasminogen to plasmin (curve IIa, tPA; Figure 5A−C); the midpoint of fibrinolysis was between 6 and 7 min in each case in the absence of a fibrinolytic inhibitor. All three agents inhibited fibrinolysis in a dose-dependent manner. Max OD405, OD405 at 60 min and the time to reach fibrinolysis midpoint at each concentration of the inhibitor used are provided in Table 2. Max OD405 did not differ between the inhibitors or with different concentrations of inhibitor. Max OD405 reflected the IIa-induced strength of the fibrin clot formed, which was achieved rapidly before subsequent lysis commenced by tPA generated plasmin at the clot site. Thus, it was anticipated that max OD405 at different concentrations of each inhibitor used would be similar. Further, OD405 at 60 min indicated the extent of fibrinolysis, which was relatively similar for each inhibitor at lower concentrations; however, it was more reduced for KD1-L17R-K_COOH_ and moderately reduced for KD1-Y11T/L17R-K_COOH_ as compared to aprotinin at higher concentrations (Figure 5, Table 2).

**Figure 6:**
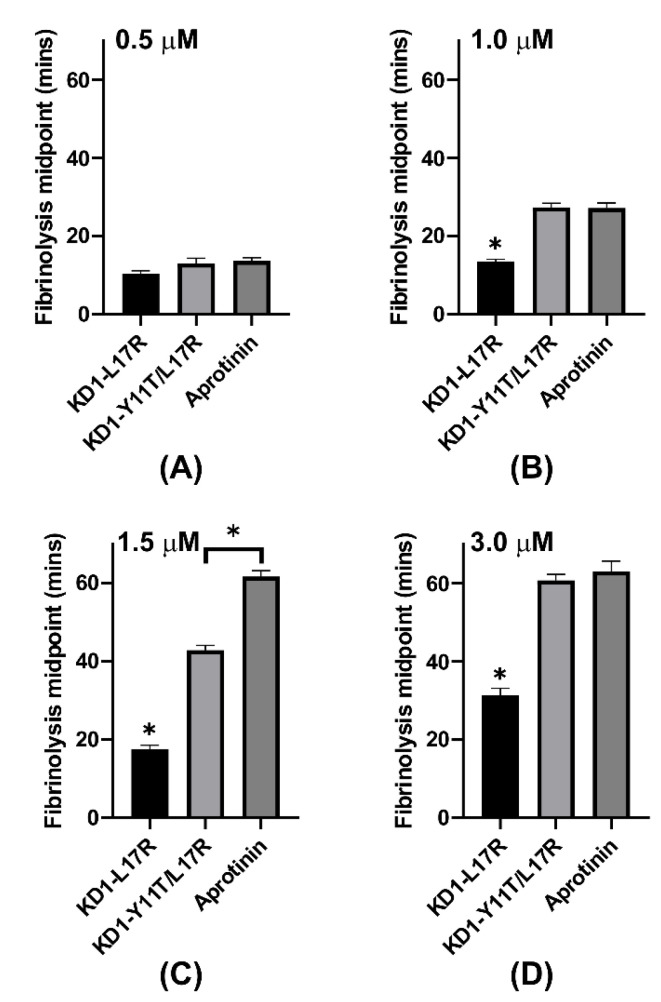
Comparison of the fibrinolysis midpoints for KD1-L17R-K_COOH_, KD1-Y11T/L17R-K_COOH_ and aprotinin at various concentrations used ((**A**), 0.5 μM; (**B**), 1.0 μM; (**C**), 1.5 μM; and (**D**), 3.0 μM) in the plasma clot lysis assay. Bar graphs are presented displaying time (minutes) to reach fibrinolysis midpoints with KD1-L17R-K_COOH_, KD1-Y11T/L17R-K_COOH_ and aprotinin at the indicated concentration. Concentration of each inhibitor used is indicated for each panel. All experiments were performed in triplicate and the mean ± SD values are presented. Note: The * without bar represents significant difference from all other agents listed. The * indicates *p* < 0.05.

Importantly, KD1-L17R-K_COOH_ increased the fibrinolysis midpoint from ~7 min to ~10 min at 0.5 µM, ~13 min at 1 µM, ~17 min at 1.5 µM, ~31 min at 3 µM, ~43 min at 4 µM and ~55 min at 5 μM, respectively (Figure 5A, Table 2). KD1-Y11T/L17R-K_COOH_ increased the fibrinolysis midpoint from ~7 min to ~12 min at 0.5 µM, ~28 min at 1 µM, ~43 min at 1.5 µM and > 60 min at 2 µM, as well as at 3 µM, respectively (Figure 5B, Table 2). Aprotinin increased the midpoint of fibrinolysis from ~7 min to ~13 min at 0.5 µM, ~40 min at 1 µM, and > 60 min at 1.5 µM as well as at >1.5 µM concentration, respectively (Figure 5C, Table 2). Cumulatively, the statistical analyses presented in Figure 6 reveal that KD1-Y11T/L17R-K_COOH_ was more effective in increasing the fibrinolysis midpoint as compared to KD1-L17R-K_COOH_, and aprotinin was slightly more effective than KD1-Y11T/L17R-K_COOH_.

### 3.6. Thromboelastography

**Figure 7:**
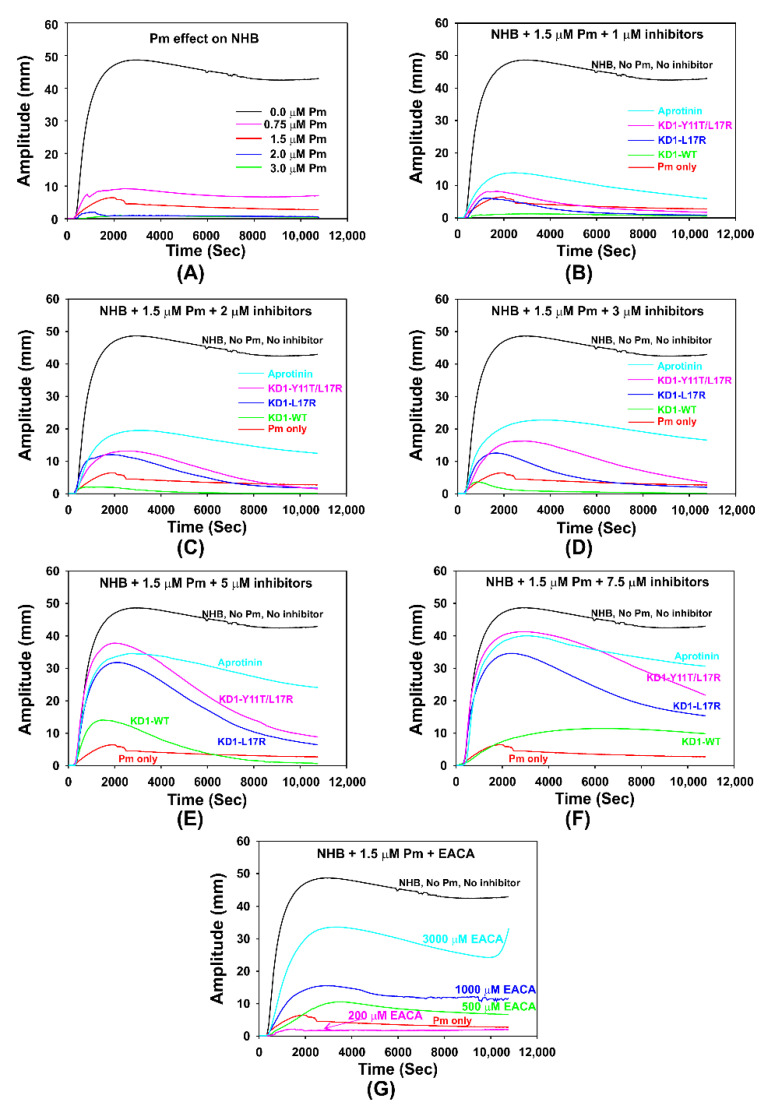
Thromboelastograms illustrating the dose-response analysis of KD1-WT, KD1-L17R-K_COOH_, KD1-Y11T/L17R-K_COOH_, aprotinin and EACA. All experiments contained citrated whole blood (300 µL), 1.5 µM plasmin and 10 mM CaCl2. The antifibrinolytic agent was added first to the blood, which was then spiked with 1.5 µM plasmin and 10 mM CaCl_2_. The clot formation and lysis were monitored for 180 min. Control experiments were performed in the presence or absence of plasmin without any antifibrinolytic agent. (**A**) Plasmin effect of clot formation and fibrinolysis. Citrated whole blood (300 µL) was spiked with various concentrations of plasmin (0–3 µM) and 10 mM CaCl_2_. The clot formation and lysis was monitored for 180 min. Effect of 1 µM (**B**), 2 µM (**C**), 3 µM (**D**), 5 µM (**E**) and 7.5 µM (**F**) of KD1-WT, KD1-L17R-K_COOH_, KD1-Y11T/L17R-K_COOH_ and aprotinin on clot formation and fibrinolysis using 1.5 µM plasmin. (**G**) Effect of 200 µM to 3000 µM EACA on clot formation and fibrinolysis using 1.5 µM plasmin. Pm, plasmin; NHB, Normal human blood.

Thromboelastography experiments were performed to evaluate the effect of KD1-WT [31], KD1-L17R-K_COOH_, KD1-Y11T/L17R-K_COOH_, aprotinin and EACA on the plasmin induced lysis of clot formed in whole blood by the addition of CaCl_2_. These data are presented in Figure 7 and summarized in Table 3. Figure 7A shows the TEG traces at different concentrations of plasmin on the clot formation initiated with CaCl_2_. In the absence of plasmin, the average maximal amplitude (MA) achieved was ~47 mm with a shear elastic modulus strength G of ~4620 dyn/cm^2^, and no clot lysis could be detected at 60 min (LY60 < 0.1%). At 1.5 µM plasmin, the MA reached was ~7 mm with a G value of ~401 dyn/cm^2^ and 100% clot lysis occurred within 30 min (Table 3). At >1.5 µM plasmin, no clot formation was observed. Figure 7B–F illustrate the average TEG traces at different concentrations (1 µM to 7.5 µM) of KD1-WT, KD1-L17R-K_COOH_, KD1-Y11T/L17R-K_COOH_ and aprotinin on clot formation and lysis in the presence of 1.5 µM plasmin. The data indicate that all antifibrinolytics tested improved the clot firmness (MA) and shear strength (G) and inhibited fibrinolysis in a concentration dependent manner (Table 3). Notably, at inhibitor concentrations of 5 µM (corresponding to the high dose of the Hammersmith regime, which is the established clinical administration regimen for aprotinin) [33], KD1-Y11T/L17R-K_COOH_ improved the clot strength MA to ~80% (37.5 mm) and G to ~65% (~3004 dyn/cm^2^, whereas aprotinin improved the MA to ~69% (32.9 mm) and G to ~53% (~2453 dyn/cm^2^). However, LY60 of ~12% was observed with KD1-Y11T/L17R-K_COOH_ compared to 0.2% with aprotinin. At 7.5 µM concentration, both KD1-Y11T/L17R-K_COOH_ and aprotinin had similar MA (~83% and ~80%) and G (~70% and ~65%), as well as LY60 (each 0.2%). EACA also improved the MA, G and LY60 in a dose dependent manner (Figure 7G). However, at 3-mM concentration of EACA, i.e., the dose used in the clinical setting, it improved the MA and G only up to ~67% and ~50% respectively. Cumulatively, the TEG data indicate that EACA is not as effective as KD1-Y11T/L17R-K_COOH_ or aprotinin in restoring the MA and G. Furthermore, KD1-WT and KD1-L17R-K_COOH_ were also not as effective as KD1-Y11T/L17R-K_COOH_ or aprotinin. Importantly, at higher concentrations (≥7.5 µM), KD1-Y11T/L17R-K_COOH_ restored the MA and G, and inhibited fibrinolysis similar to aprotinin.

**Figure 8:**
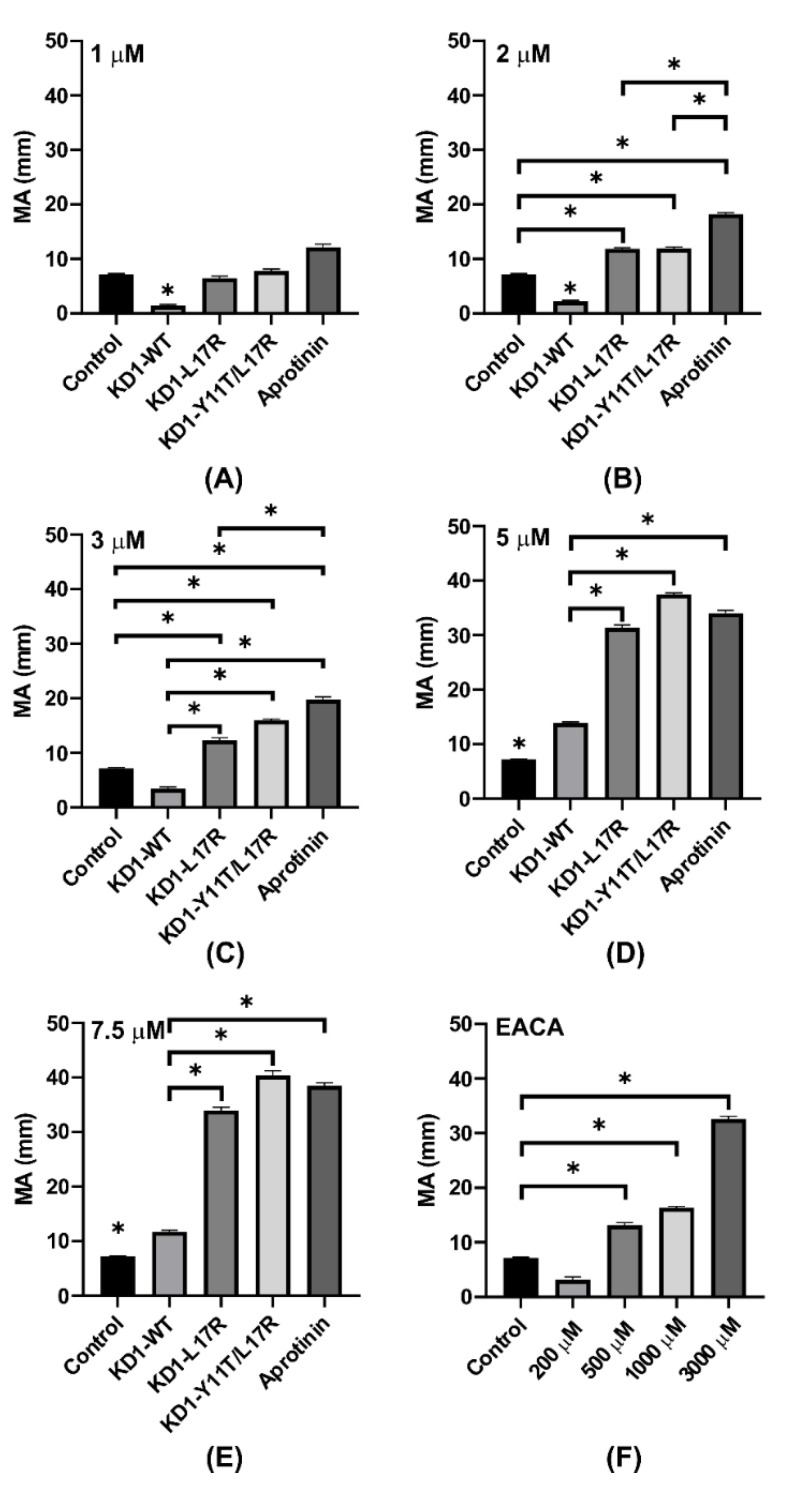
Comparison of maximal amplitude (MA) from the TEG experiments for KD1-WT, KD1-L17R-K_COOH_, KD1-Y11T/L17R-K_COOH_, aprotinin and EACA at different concentrations. Bar graphs represent the MA achieved with KD1-WT, KD1-L17R-K_COOH_, KD1-Y11T/L17R-K_COOH_, aprotinin and EACA at different concentrations. Panel (**A**), 1 µM; panel (**B**), 2 µM; panel (**C**), 3 µM; panel (**D**), 5 µM; panel (**E**), 7.5 µM; and panel (**F**), 200 μM to 3000 μM EACA. All experiments were performed in duplicate and the mean ± SD values are presented. Note: The * without bar represents significant difference from all other agents listed. The * indicates *p* < 0.05.

**Figure 9:**
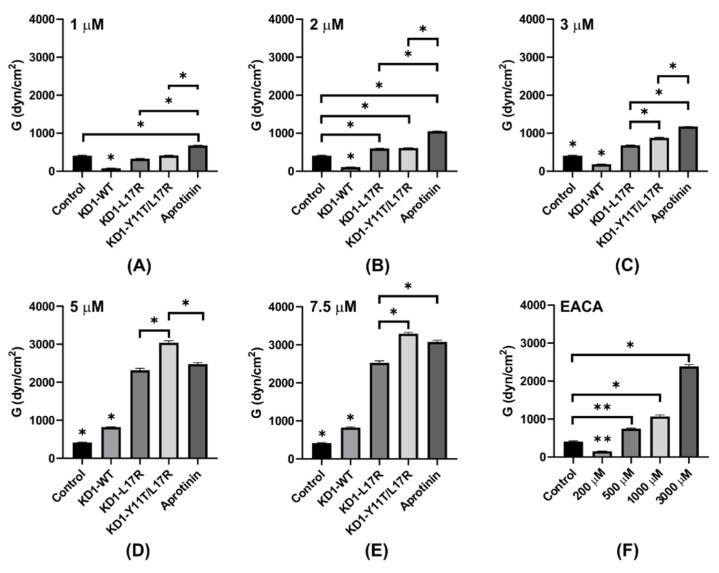
Comparison of shear elastic modulus strength (G, TEG experiments) for KD1-WT, KD1-L17R-K_COOH_, KD1-Y11T/L17R-K_COOH_, aprotinin and EACA at different concentrations. Bar graphs represent the ‘G’ achieved with KD1-WT, KD1-L17R-K_COOH_, KD1-Y11T/L17R-K_COOH_, aprotinin and EACA at different concentrations. Panel (**A**), 1 µM; panel (**B**), 2 µM; panel (**C**), 3 µM; panel (**D**), 5 µM; panel (**E**), 7.5 µM; and panel (**F**), 200 μM to 3000 μM EACA. All experiments were performed in duplicate and the mean ± SD values are presented. Note: The * without bar represents significant difference from all other agents listed. The * indicates *p* < 0.05 and ** indicates *p* < 0.01.

**Figure 10:**
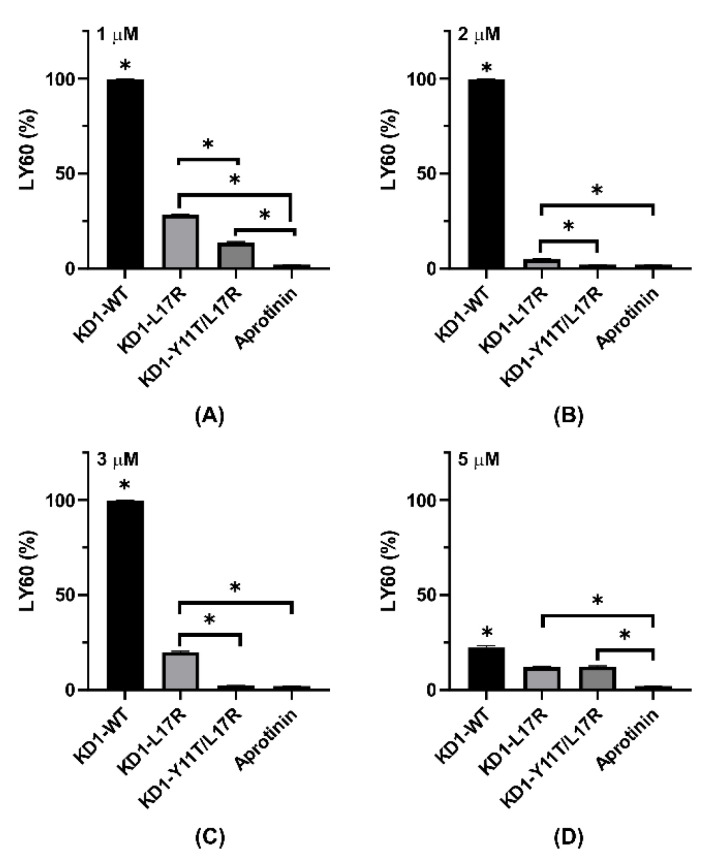
Comparison of LY60% (TEG experiments) for KD1-WT, KD1-L17R-K_COOH_, KD1-Y11T/L17R-K_COOH_ and aprotinin at different concentrations. Bar graphs showing the percent lysis at 60 min are depicted. Panel (**A**), 1 μM; panel (**B**), 2 μM; panel (**C**), 3 μM; and panel (**D**), 5 μM. All experiments were performed in duplicate and the mean ± SD values are presented. Note: The * without bar represents significant difference from all other agents listed. The * indicates *p* < 0.05.

Multiple comparison analyses performed on the concentration-dependent enhancement of maximal amplitude (MA), shear elastic modulus strength (G) and LY60 by KD1-WT, KD1-L17R-K_COOH_, KD1-Y11T/L17R-K_COOH_ and aprotinin in TEG experiments are presented in Figure 8, Figure 9 and Figure 10. At 1 µM inhibitor concentration, the MA was not significantly different between the control and each Kunitz inhibitor except the KD1-WT (Figure 8A). At 2 or 3 µM inhibitor concentrations, the MA enhancement by aprotinin was statistically significant (*p* < 0.05) as compared to KD1-WT, KD1-L17R-K_COOH_ and KD1-Y11T/L17R-K_COOH_ (Figure 8B,C). Above 3 µM, enhancement in MA was statistically not different for KD1-L17R-K_COOH_, KD1-Y11T/L17R-K_COOH_ and aprotinin; however, it was significantly lower for KD1-WT (Figure 8D,E). For EACA, Student’s t-test was performed to compare MA between control and each EACA concentration tested (Figure 8F). At 500 µM, 1000 µM or 3000 µM EACA concentration, the MA enhancement was statistically significant as compared to the control.

Notably up to 3 µM, aprotinin enhanced G significantly compared to the KD1-based inhibitors (Figure 9A–C). Surprisingly, at 5 and 7.5 µM inhibitor concentrations, the enhancement of G by KD1-Y11T/L17R-K_COOH_ was significantly higher as compared to the other inhibitors (Figure 9D,E). This observed improvement in clot shear strength G for KD1-Y11T/L17R-K_COOH_ versus aprotinin might possibly have been to FXIa and kallikrein inhibition by aprotinin versus essentially no inhibition by KD1-Y11T/L17R-K_COOH_. Additionally, multiple comparison analyses of LY60 for each inhibitor at selected concentrations are presented in Figure 10. At 1 or 5 µM, aprotinin was significantly better at preventing fibrinolysis compared to each KD1 inhibitor, whereas KD1-WT was inferior to each inhibitor at all concentrations tested. At 2 or 3 µM, KD1-Y11T/L17R-K_COOH_ and aprotinin were superior to KD1-L17R-K_COOH_ and no LY60 was observed with any inhibitor at 7.5 µM concentration. Overall, it would appear that aprotinin and KD1-Y11T/L17R-K_COOH_ were superior to other inhibitors at inhibiting fibrinolysis in the TEG experiments.

### 3.7. Cell Toxicity Studies

Here, we wanted to gain insights into the potential toxicity of KD1-Y11T/L17R-K_COOH_ compared with aprotinin and the currently used antifibrinolytic agents EACA and TXA. Patients are typically treated with antifibrinolytic agents via intravenous injections while undergoing major surgery, or via external use in trauma situations. We therefore tested cytotoxicity in endothelial cells and skin fibroblasts, the cells most likely to be exposed to therapeutic doses of KD1-Y11T/L17R-K_COOH_. The plasma half-life of TXA in humans, rats and dogs is ~120 min [48]. The half-life each of the two KD1 variant homologs (aprotinin and Ecallantide) is also ~120 min [49,50] in humans, whereas the half-life of aprotinin in mice, rats or dogs is ~70 min [51]. The half-life of each KD1 variant is not known, but might be short, and will be determined in future research. Since the half-life of each of the antifibrinolytic agents in vivo is short, infusion is usually continuous throughout the duration of surgery. Treatment duration was therefore set at 24 h and the chosen dose range included the equivalent of ~3x the clinical dose for each of the reagents tested.

**Figure 11:**
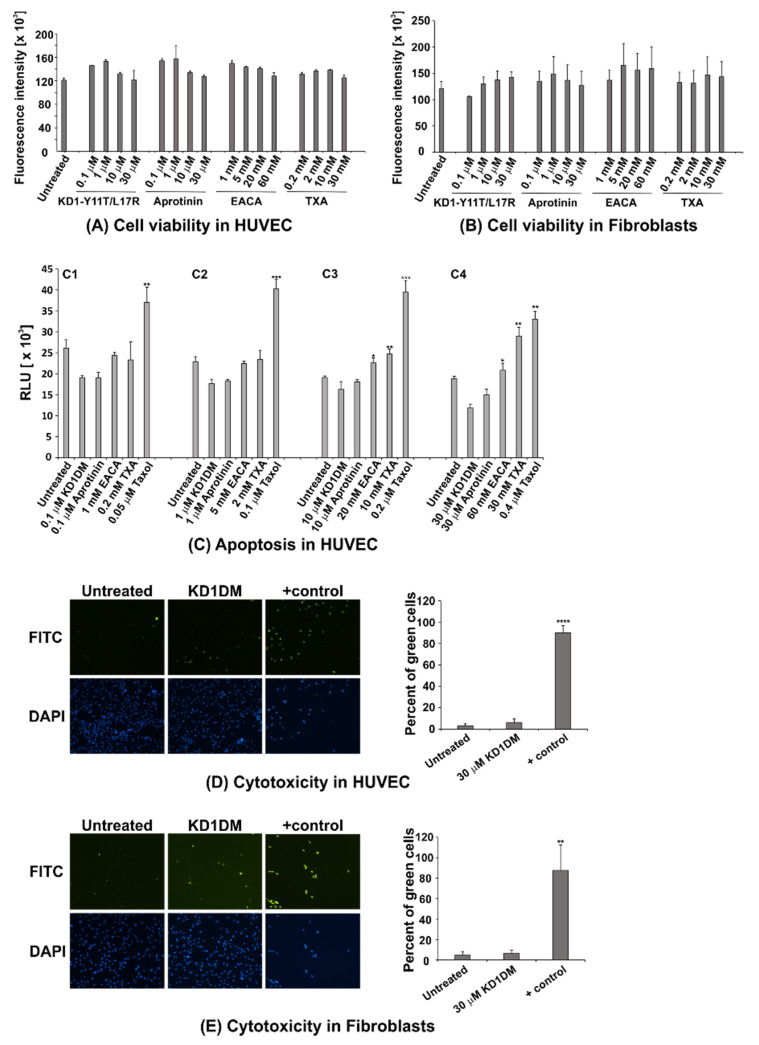
Toxicity studies of KD1-Y11T/L17R-K_COOH_, aprotinin, EACA and TXA in primary endothelial cells or fibroblasts. (**A**) Cell viability in HUVEC and (**B**) in primary human skin fibroblasts. Cells were treated with KD1-Y11T/L17R-K_COOH_, aprotinin, EACA, or TXA for 24 h at the indicated concentrations. Fluorescence intensity is plotted against concentration of each antifibrinolytic inhibitor. Note that, fluorescence intensity is proportional to relative cell number. Data points represent means from three independent experiments ± SEM. In each case, cell viability appears to be not significantly different from the untreated cells (*p* > 0.05). (**C**) Apoptosis in HUVEC cells. HUVEC cells were either untreated or treated with KD1-Y11T/L17R-K_COOH_, aprotinin, EACA or TXA for 24 h at increasing concentrations (**C1**–**C4**). Taxol was included as a positive control. Luminescence, displayed as relative light units (RLU), is proportional to caspase-3/7 activity. EACA and TXA, but not KD1-Y11T/L17R-K_COOH_ or aprotinin show significantly increased caspase activity at concentrations used in C3 and C4 compared to the untreated cells. Data are mean ± SD from three experiments. (* *p* < 0.05, ** *p* < 0.01, *** *p* < 0.001). (**D**) Absence of cytotoxicity in HUVEC and (**E**) primary human skin fibroblasts with KD1-Y11T/L17R-K_COOH_. Cells were either untreated or treated with 30 µM KD1-Y11T/L17R-K_COOH_ for 24 h. Taxol (0.05 µM) was included as positive control. FITC: CellTox green dye binds to DNA when membrane integrity has been compromised. Fluorescent signal indicates cytotoxicity. DAPI: nuclear stain, binds to all nuclei. Representative images of one of three independent experiments performed are shown. Graph depicts quantification of cytotoxicity assay. Percent of green cells out of all cells per field were calculated. Mean ± SD value of 4 fields per treatment group are displayed (** *p* < 0.01, **** *p* < 0.0001).

A resazurin assay of HUVEC treated with KD1-Y11T/L17R-K_COOH_ or aprotinin for 24 h did not result in any significant change in cell viability compared to cells treated with phosphate buffer control over the entire dose range from 0.1–30 µM (Figure 11A). The same result was obtained after treatment with EACA (dose range 1–60 mM) and TXA (dose range 0.2–30 mM). Cell viability was equally unchanged in primary human skin fibroblasts (Figure 11B), indicating that none of the antifibrinolytic agents tested caused measurable cytotoxicity within the 24 h duration of treatment.

Viability is the endpoint of cytotoxicity. Thus, we examined the induction of apoptosis resulting from caspase activation. Caspase 3/7 assays were performed in HUVEC cells following treatment with antifibrinolytic agents (Figure 11C). Caspase 3/7 activities significantly increased when the cells were treated with the two higher concentrations of TXA (10 mM and 30 mM) and, to a lesser extent, after exposure with EACA (20 mM and 60 mM). In contrast to TXA and EACA, KD1-Y11T/L17R-K_COOH_ and aprotinin did not induce caspase activity above baseline at all concentrations. Taxol was included as a positive control. None of the antifibrinolytics increased caspase activity above baseline in primary fibroblasts across all doses.

To confirm the above results using a different assay, we performed CellTox green cytotoxicity assays in HUVEC cells and primary fibroblasts. The CellTox green dye binds DNA, resulting in fluorescent staining only when membrane integrity has been compromised. No significant increase in the percentage of fluorescent cells could be detected 24 h after treatment with the highest dose (30 µM) of KD1-Y11T/L17R-K_COOH_ in the endothelial cells (Figure 11D) or in the fibroblasts (Figure 11E). Cell cytotoxicity also did not increase significantly over baseline when cells (HUVEC or fibroblasts) were treated with other antifibrinolytic agents. For brevity, the data for aprotinin, TXA and EACA are not shown.

In summary, 24 h treatment of HUVEC cells and primary human fibroblasts with 0.1–30 µM KD1-Y11T/L17R-K_COOH_ or aprotinin did not decrease viability, induce apoptosis or show any sign of cytotoxicity. However, TXA and EACA induced apoptosis (cell death) at higher concentrations in HUVEC cells, as inferred from an increase in caspase 3/7 activity.

## 4. Discussion

Earlier, based on structural information and S2′-subsite specificity, we designed a 73-residue Kunitz domain plasmin inhibitor from TFPI-2 KD1 [32]. The KD1-WT inhibits plasmin as well as pKLK, FXIa and FVIIa/TF with comparable affinities, whereas KD1-L17R inhibits only plasmin. The change in residue 17 (BPTI numbering) from Leu to Arg made the KD1-L17R specific for plasmin and dramatically reduced pKLK and FXIa inhibition. As compared to the current 60-residue KD1-L17R-K_COOH_, the previously expressed KD1-L17R had 13 additional residues (9 from the TFPI-2 sequence and 4 from the IIa cleavage site) at the N-terminus and four (VPKV) at the C-terminus, apart from the core Kunitz domain. Although these additional residues do not interfere with KD1-L17R function, they are flexible and could be disordered as inferred from the crystal structure of the KD1-WT [36]. 

Therefore, a new 60-residue KD1-L17R-K_COOH_ mutant was expressed and its inhibition profile was characterized. Since none of the active site inhibition profiles of 60-residue KD1-L17R-K_COOH_ had changed from the previously expressed 73-residue KD1-L17R, it was predicted that KD1-L17R-K_COOH_ would be very effective in reducing blood loss and could be comparable to aprotinin in the two mouse bleeding models (liver laceration and tail-amputation) tested [32,41,52].

**Figure 12:**
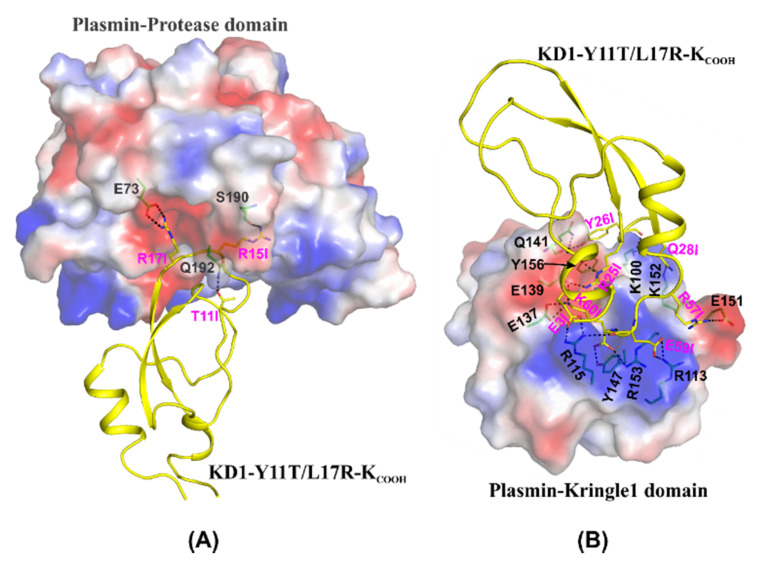
Modeled complexes of KD1-Y11T/L17R-K_COOH_ interaction with plasmin. (**A**) Modeled interactions of KD1-Y11T/L17R-K_COOH_ with the plasmin protease domain. The electrostatic surface of the plasmin protease domain and a cartoon representation of the KD1-Y11T/L17R-K_COOH_ (yellow) are depicted. The P1 (Arg15), P5 (Thr11) and P2′ (Arg17) residues of KD1-Y11T/L17R-K_COOH_ interactions with plasmin are shown in stick representation. In the electrostatic surface, blue represents positive, red represents negative, and white represents neutral charge. (**B**) Modeled interaction of KD1-Y11T/L17R-K_COOH_ with plasmin kringle domain. The electrostatic surface of the plasminogen kringle domain1 and a cartoon representation of the KD1-Y11T/L17R-K_COOH_ (yellow) are depicted. The residues that form hydrogen bonds and salt bridges (shown as dashed lines) between the kringle domain and KD1-Y11T/L17R-K_COOH_ are shown in stick representation. The carbon atoms are shown in green for the kringle domain and yellow for KD1-Y11T/L17R-K_COOH_. As in (**A**) oxygen atoms are shown in red and nitrogen atoms in blue. The KD1-Y11T/L17R-K_COOH_ residues are labeled with the suffix I. In the electrostatic surface, blue represents positive, red represents negative, and white represents neutral charge.

The 73-residue KD1-L17R has IEKVPKV at the C-terminus and valine could be removed by extended incubation with IIa [41]. The removal of Val residue at the C-terminus generated a C-terminal lysine that made the KD1-L17R a dual reactive inhibitor of fibrinolysis by inhibiting the plasmin active site, as well as plasminogen activation [41]. Moreover, extended incubation with IIa resulted in a heterogeneous population of KD1-L17R with different N-terminal residues [41]. The structural analysis of the modeled complex of plasmin and KD1-L17R indicated that changing residue Tyr11 to Thr would be beneficial for plasmin inhibition. Threonine in KD1-Y11T/L17R-K_COOH_ made an additional hydrogen bond with residue Q192 of plasmin (Figure 12A). Interestingly, 73-residue KD1-L17R contained two lysine residues at the C-terminal segment (IEKVPKV), and either of them could serve as a C-terminal residue. Further, the modeling of 60-residue KD1-L17R-K_COOH_ with the C-terminus IEK sequence showed that it will enhance the interactions with the kringle domains of plasminogen and tPA (Figure 12B). Compared to the VPK sequence, the IEK sequence had two additional interactions arising from Arg57 and Glu59 of Kunitz domain with plasmin kringle residues Glu151 and Arg153, respectively (Figure 12B). Similar interactions are predicted to occur with the kringle domain of tPA as well. For these reasons, the 60-residue double mutant (KD1-Y11T/L17R-K_COOH_) was expressed with the IEK C-terminus.

The newly *E. coli* expressed KD1-L17R-K_COOH_ and KD1-Y11T/L17R-K_COOH_ with C-terminal IEK sequence both contained His6-tag and the enterokinase cleavage sequence; however, these additional residues could not be removed by enterokinase. Similar to the 73-residue KD1-L17R construct, the presence of additional residues did not affect the inhibition properties of KD1-L17R-K_COOH_ and KD1-Y11T/L17R-K_COOH_ mutants. Therefore, the 60-residue KD1-Y11T/L17R-K_COOH_ was expressed in *P. pastoris*. As predicted, KD1-Y11T/L17R-K_COOH_ inhibited plasmin with increased affinity as compared to KD1-L17R-K_COOH_ (0.59 nM vs. 0.9 nM). Further, the 60-residue KD1-Y11T/L17R-K_COOH_ with IEK C-terminal bound to the kringle domains of tPA and plasmin with increased affinity (35 nM to 50 nM) Figure 5 as compared to the KD1-L17R-K_T_ with C-terminal VPK (250 nM to 300 nM) [41]. The modest increase in plasmin active site inhibition and significantly improved affinity for kringle domains of plasminogen and tPA was reflected in strong inhibition of fibrinolysis by KD1-Y11T/L17R-K_COOH_ in plasma clot lysis assay (Figure 4B) and in restoring MA, G and LY60 in the TEG experiments (Figure 7B–F).

The KD1 double mutant (KD1-Y11T/L17R-K_COOH_) made in *P. pastoris* is a compact, homogeneous and an effective specific plasmin inhibitor of human origin. The properties of KD1-Y11T/L17R-K_COOH_ were comparable to aprotinin in plasmin inhibition assay, plasma clot lysis assay and in the TEG experiments. Moreover, KD1-Y11T/L17R-K_COOH_ did not inhibit pKLK, FXIa and FVIIa/sTF. Furthermore, KD1-Y11T/L17R-K_COOH_ did not induce any measurable cytotoxicity in primary endothelial cells or skin fibroblasts (Figure 11). However, TXA and EACA caused apoptosis in these cells at higher concentrations, which could be achieved during renal clearance of these antifibirinolytics. These results are in agreement with KD1-L17R-K_T_ single mutant, which did not induce renal toxicity, seizures or any detectable histopathologic changes in the mouse kidney [32]. In case of aprotinin, its acidic nature and pKLK inhibition results in altered renal activity, which leads to kidney damage [32,53]. The current antifibrinolytics EACA and TXA cause seizures by inhibiting glycine receptors [54]. Since lysine analogs are not as effective as aprotinin, the higher doses of EACA and TXA increase the risk of renal failure, as these agents reach very high concentrations during clearance by glomerular filtration [55,56]. The KD1Y11T/L17R-K_COOH_ data from the current study are encouraging; however, the compound needs to be evaluated in suitable animal bleeding models before it can be considered for clinical trials.

## 5. Patents

S.P. Bajaj has a patent pending on the KD1-L17R and related molecules.

## Figures and Tables

**Table 1:** *K_i_* values for inhibition of plasmin by KD1-WT, KD1-Y11T-K_COOH,_ KD1-L17R/Y11T-K_COOH_ and aprotinin.

Inhibitor	*K_i_* (nM) *
KD1-WT	6 ± 0.5
KD1-L17R-K_COOH_	0.9 ± 0.1
KD1-Y11T/L17R-K_COOH_ (*E. coli*)	0.59 ± 0.1
KD1-Y11T/L17R-K_COOH_ (*Pichia*)	0.59 ± 0.1
Aprotinin	0.49 ± 0.1

* *K_i_* values represent an average ± SD of three independent measurements.

**Table 2:** The effect of KD1-L17R-K_COOH_, KD1-Y11T/L17R-K_COOH_ and aprotinin on plasma clot lysis.

Inhibitor Concentration (µM)	Max OD405			OD405 at 60 min			Fibrinolysis Midpoint Time (Minutes)		
	KD1 L17R-K_COOH_	KD1 Y11T/L17R- K_COOH_	Aprotinin	KD1 L17R- K_COOH_	KD1 Y11T/L17R- K_COOH_	Aprotinin	KD1 L17R- K_COOH_	KD1 Y11T/L17R- K_COOH_	Aprotinin
0 + No tPA	1.55 ± 0.10	1.69 ± 0.13	1.64 ± 0.14	1.49 ± 0.11	1.63 ± 0.11	1.60± 0.21	>60	>60	>60
0 + tPA	1.47 ± 0.15	1.42 ± 0.17	1.48 ± 0.20	0.88 ± 0.15	0.86 ± 0.12	0.86 ± 0.11	7 ± 1	7 ± 1	7 ± 1
0.5 + tPA	1.48 ± 0.11	1.54 ± 0.14	1.54 ± 0.15	0.85 ± 0.14	0.85 ± 0.13	0.85 ± 0.10	10 ± 0.76	13 ± 1.25	13 ± 0.8
1.0 + tPA	1.56 ± 0.10	1.54 ± 0.11	1.63 ± 0.13	0.87 ± 0.11	0.84 ± 0.12	1.04 ± 0.14	13 ± 0.5	27 ± 1.1	27 ± 1.5
1.5 + tPA	1.61 ± 0.13	1.58 ± 0.13	1.62 ± 0.08	0.90 ± 0.14	0.86 ± 0.15	1.22 ± 0.12	17 ± 1.0	43 ± 1.6	>60
2.0 + tPA		1.58 ± 0.09	1.62 ± 0.10		1.35 ± 0.06	1.50 ± 0.09		>60	>60
3.0 + tPA	1.74 ± 0.05	1.71 ± 0.10	1.68 ± 0.07	0.80 ± 0.13	1.57 ± 0.08	1.56 ± 0.05	31 ± 1.75	>60	>60
4.0 + tPA	1.62 ± 0.11			0.92 ± 0.05			43 ± 1		
5.0 + tPA	1.69 ± 0.08			1.13 ± 0.06			55 ± 1.5		

**Table 3:** Effect of KD1-WT, KD1-L17R-K_COOH_, KD1-Y11T/L17R-K_COOH_, aprotinin and EACA on the TEG Parameters.

Inhibitor Concentration	(NHB+1.5 µM Plasmin) Inhibitor	MA (mm)	G (dyn/cm^2^)	LY30 (%)	LY60 (%)
0 µM		7.18 ± 0.17	411 ± 13.1	100	100
1 µM	KD1-WT	1.45 ± 0.28	79.05 ± 5.7	100	100
	KD1-L17R-K_COOH_	6.45 ± 0.49	328.75 ± 9.3	10.1 ± 0.32	28.4 ± 0.49
	KD1-Y11T/L17R-K_COOH_	7.82 ± 0.45	414.3 ± 10.1	0.75 ± 0.6	13.7 ± 0.64
	Aprotinin	12.15 ± 0.78	678.6 ± 5.4	0	0.30 ± 0.08
2 µM	KD1-WT	2.23 ± 0.25	107.9 ± 3.2	100	100
	KD1-L17R-K_COOH_	11.84 ± 0.33	598.7 ± 6.1	0	4.95 ± 0.35
	KD1-Y11T/L17R-K_COOH_	11.88 ± 0.41	611.7 ± 5.8	0	0.45 ± 0.07
	Aprotinin	18.19 ± 0.43	1050.5 ± 8.3	0	0
3 µM	KD1-WT	3.49 ± 0.41	180.15 ± 7.1	100	100
	KD1-L17R-K_COOH_	12.34 ± 0.61	683.2 ± 9.8	0	19.9 ± 0.42
	KD1-Y11T/L17R-K_COOH_	16.03 ± 0.31	879.3 ± 15.6	0	2.2 ± 0.28
	Aprotinin	19.78 ± 0.68	1174.1 ± 16.7	0	0
5 µM	KD1-WT	13.90 ± 0.28	817.95 ± 10.1	6.5 ± 0.7	22.4 ± 0.92
	KD1-L17R-K_COOH_	31.35 ± 0.76	2315.15 ± 49.4	0	12.3 ± 0.50
	KD1-Y11T/L17R-K_COOH_	37.48 ± 0.40	3004.75 ± 50.6	0	12.2 ± 0.49
	Aprotinin	34.04 ± 0.77	2453.2 ± 36.9	0	0.2 ± 0.04
7.5 µM	KD1-WT	11.73 ± 0.38	820.1 ± 21.3	0	0
	KD1-L17R-K_COOH_	33.93 ± 0.88	2527.5 ± 49.6	0	5 ± 0.52
	KD1-Y11T/L17R-K_COOH_	40.37 ± 1.22	3292.45 ± 35.0	0	0.2 ± 0.03
	Aprotinin	38.45 ± 0.78	3078.1 ± 44.9	0	0.2 ± 0.02
200 µM	EACA	3.20 ± 0.71	153.25 ± 9.8	0	0
500 µM	EACA	13.15 ± 0.64	747.75 ± 14.8	0	0
1000 µM	EACA	16.40 ± 0.28	1070.1 ± 35.0	0	16.8 ± 0.35
3000 µM	EACA	32.60 ± 0.71	2388.0 ± 49.6	0	0.6 ± 0.04

NHB, Normal human blood; MA, maximal amplitude (maximal clot strength); G, shear strength; LY30, Percent lysis observed at 30 min after clot formation; LY60, Percent lysis observed at 60 min after clot formation. Mean ± SD are provided.
